# Research progress on microecology and childhood respiratory infections via the lung-gut axis

**DOI:** 10.3389/fped.2025.1509333

**Published:** 2025-06-19

**Authors:** Zhixuan He, Zixuan Wang, Jiao Yin, Qiang Wang

**Affiliations:** ^1^ Institute of Infection, Immunology and Tumor Microenvironment, Hubei Province Key Laboratory of Occupational Hazard Identification and Control, Medical College, Wuhan University of Science and Technology, Wuhan, China; ^2^ Department of Immunology, School of Basic Medicine, Hubei University of Arts and Science, Xiangyang, China

**Keywords:** gut-lung axis, pediatric respiratory tract infections (RTIs), microbiota, microecology, probiotics

## Abstract

Respiratory tract infections (RTIs) are a complex global public health challenge, with children being the most affected population. Affected children often exhibit gut microbiota-related symptoms, such as dysbiosis, feeding difficulties, and malabsorption. Studies show that the lungs and large intestine share embryological homology and a mucosal immune system, with gut microbiota influencing respiratory health—a phenomenon termed the “lunggut axis” Gut dysbiosis may disrupt respiratory microbiota homeostasis, elevating susceptibility to respiratory infections. Probiotic administration mitigates gut dysbiosis and antibiotic resistance induced by antibiotic overuse. It can also restore the balance of gut microbiota, enhance the immune response and metabolic regulation, and thus prevent and assist in treating respiratory infections and other respiratory diseases. A deeper understanding of the relationship between the lung-gut axis microecology and respiratory infections in children may provide novel insights and approaches for disease prevention, diagnosis, and treatment. This review will describe the normal microecology of the respiratory tract in children, the microecological changes associated with respiratory tract infections in children, and the interactions between the “lung-gut axis” and the use of probiotics. It will also provide an outlook on these topics.

## 1 Introduction

Symbiotic microorganisms colonize the human body's surfaces and externally connected cavities (e.g., skin, vagina, intestines), outnumbering human cells by 10-fold. However, respiratory microbiota research remains nascent. The gut microbiota, comprising ∼40 trillion microorganisms, is one of the largest human microbial communities. Its gene pool is ∼150 times larger than the human genome, earning it the designation “enteric nervous system” or “second brain” (1). Research shows that the lung and large intestine have embryological homology, a common mucosal immune system, secretion function, and other modern biological basis, gut microbiota can regulate the function of the gastrointestinal tract but also affect respiratory health, the formation of “intestine-lung axis” (2), therefore, many scholars are researching to improve the gut microbiota and other micro-ecological way of treating or assisting in the treatment of respiratory diseases, and has achieved some results. Respiratory microecology in early life by genetic, environmental, and other factors, and through the body's metabolism, inflammation, and immune system effects on disease development and regression (3). Respiratory infections are one of the most common diseases in children, and changes in the abundance and species of respiratory and gut microbiota may occur at the onset of diseases. Probiotics such as lactobacilli are useful in the treatment of respiratory infections in children, but more research is needed. Therefore, this paper summarises recent studies in the hope of elucidating the relationship between lung and gut microbiota and respiratory infections in children, and to provide a basis for further research. We illustrated the connection between lungs and intestines during respiratory infection.

## 2 Interaction between the “lung-intestinal axis”

Differences in the mother's mode of delivery also influence infant microbiome development. Vaginal delivery exposes the infant to the mother's vaginal and gut microbiome, potentially allowing key microbiomes to become established early in the infant's colon. In contrast, cesarean delivery exposes the skin and environmental microbiome, creating significant microbial acquisition, which promotes a less desirable microbial community and affects the gut microbiome in children up to age 6. Studies have shown that children born by cesarean section are at a higher risk of mucosal immune system-related diseases than children born vaginally. This is because newborns delivered vaginally have a variety of gram-positive bacteria transferred from their mothers (e.g., Bifidobacterium bifidum), whereas newborns delivered by cesarean section do not. According to experiments in mice, intranasal exposure to Gram-negative and Gram-positive bacteria results in the synthesis of more lipopolysaccharides (LPS), which polarises the immune response to Th1 cell-mediated immune responses (4). Also, primary immune cells isolated from vaginally delivered infants produce higher levels of tumour necrosis factor (TNF-a) and interleukin 18 (IL-18) when stimulated, which may help neonates to better control respiratory viral infections through a mature immune system (5). Beyond delivery mode, the source of respiratory pathogens is multifactorial. Studies suggest that maternal transmission, environmental exposure (e.g., airborne particles), and dysbiosis-induced virulence of commensals collectively contribute to pathogenic colonization (6). Children grow up in an exposed environment, constantly receiving stimulation from external antigens, and the immune system constantly produces antibodies to prevent infections. Symbiotic microorganisms, such as respiratory microbiota and gut microbiota, have the function of regulating immunity, and play an important role in the development and work of the immune system. Respiratory microecology changes when respiratory tract infections (RTIs) occur in children, and the gut microbiota also changes to some extent in some children. Gut microbiota influences the systemic immune response, including the lungs, by regulating the maturation and function of the intestinal immune system. Gut microbiota can affect the integrity of the intestinal mucosal barrier and promote or inhibit the production of inflammatory factors, which reach the lungs with the blood circulation and participate in the immune regulation and inflammatory response in the lungs. In addition, gut microbiota can indirectly regulate the immune status of the lungs by influencing the differentiation and function of immune cells such as T cells, B cells, and dendritic cells. At the same time, gut flora are involved in the metabolism and absorption of a wide range of nutrients, including the production of SCFAs. These metabolites can influence physiological processes such as energy metabolism, fat storage, and insulin resistance in the host, and may also reach the lungs through the blood circulation and have an impact on lung cell metabolism and function. In addition, gut flora can influence the metabolism of bile acids, which act as signaling molecules and play an important role in regulating inflammation and fibrosis in the lungs. Research has shown that there is close two-way communication between gut flora and the nervous system, known as the “gut-brain axis”. However, the function of this axis is not limited to the brain, but also extends to the lungs. Gut flora can influence the activity of lung cells by affecting the release of neurotransmitters from the vagus nerve, such as acetylcholine Ach, which regulates the neural reflexes and autonomic functions of the lungs, thus affecting respiratory movements, airway tone, and airway responsiveness (7, 8). Gut microbiota can produce a variety of hormone-like substances and neurotransmitters, such as serotonin, dopamine, GABA (γ-aminobutyric acid), etc., which are able to enter the blood circulation and affect the function of several organs throughout the body, including the lungs. In addition, gut microbiota can indirectly affect the physiological function of the lungs by regulating the host's endocrine system (e.g., thyroid, adrenal gland, etc.). Traditional Chinese Medicine (TCM) also believes that the lungs and the large intestine are mutually exclusive, lung diseases and intestines, intestinal diseases and lungs, and the lungs and intestines interact with each other through the elevation of energy and the metabolism of sweat and fluids (9). Based on the study of children's gut microbiota and immune system development, Budden et al. proposed the theory of the “lung-intestinal axis” that lungs and intestines rely on embryonic homology, immune channels, neural channels and other pathways to be interconnected, which provides a new way of thinking for the treatment of children's respiratory tract infections (2).

**Figure 1:**
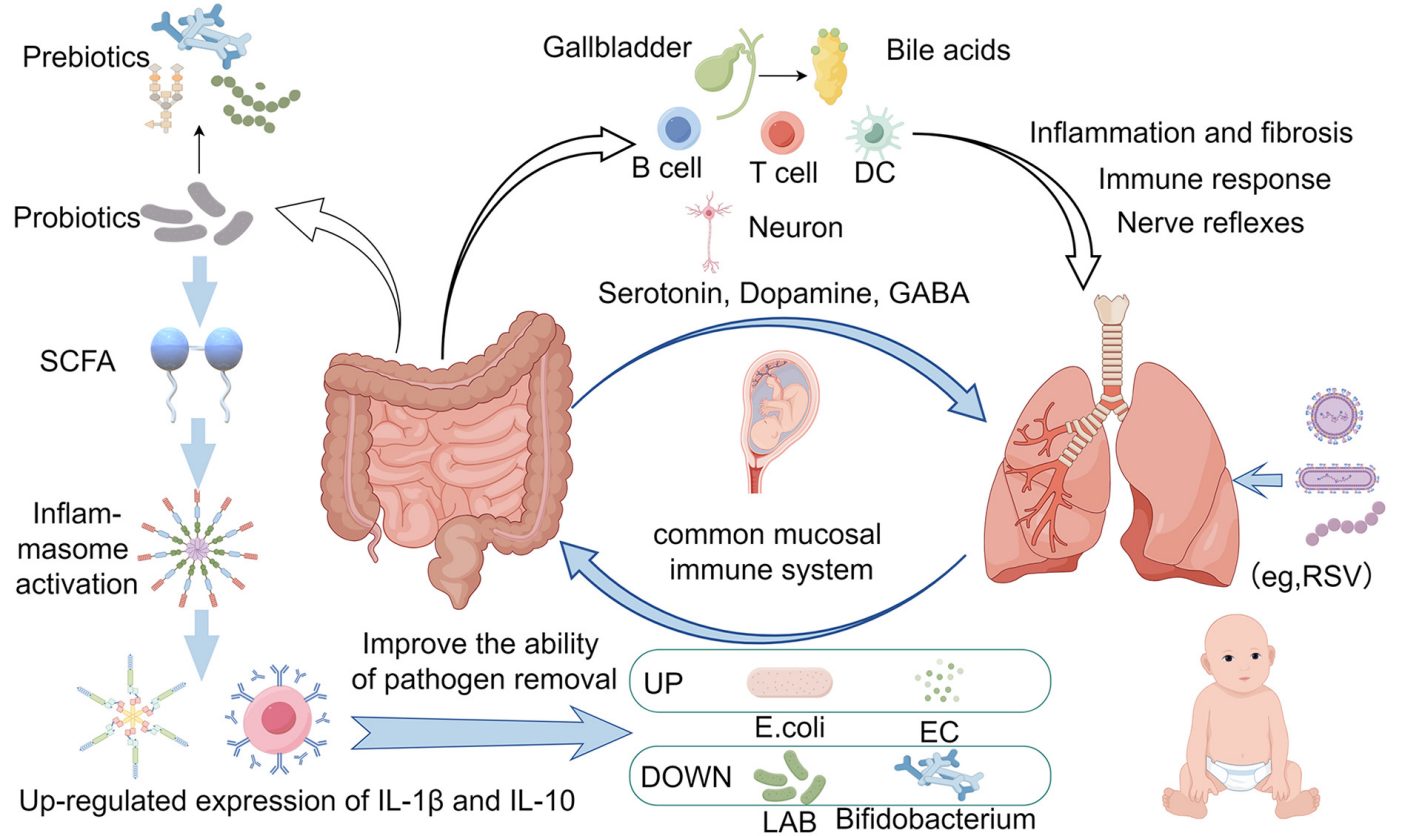
The connection between intestinal microbiota changes and pulmonary infection immunity. (Created with Figdraw).

Related studies also show that the gut microbiota can regulate the body's immune function, and can directly regulate the lung immune function, at the same time respiratory system diseases will also affect the stable state of the gut microbiota, such as wheezing children in the body of bifidobacteria decreased significantly, respiratory infections in children susceptible to diarrhoea, are indicating that the children appear to have a disturbance of gut microbiota phenomenon (10). Teo et al. reported that children with upper RTIs exhibit decreased gut microbiota abundance at the phylum level (e.g., Firmicutes, Bacteroidetes); in the level of the genus, the abundance of the genus Upper Enterococcus increased greatly, the abundance of the genus Eubacterium, anabolic, fecal bacilli genus and bifidobacteria genus on the contrary, a significant decline. Gut dysbiosis is characterized by increased opportunistic pathogens (e.g., Escherichia coli, Enterococci) and reduced probiotics (e.g., Bifidobacteria, Lactobacilli) (11). Alterations in the same gut microbiota disorders have also been shown to be present in recurrent respiratory infections in pediatrics (12). A study of 16SrRNA gene sequencing of feces of infants infected with syncytial virus showed a higher abundance of S247, Clostridium, Olfactobacteriaceae, Lactobacillus, and Actinobacillus than in normal infants in moderately and severely infected children, and a decrease in Moraxobacteriaceae flora in severely syncytial virus-infected children (13). In addition to this, children with tuberculosis had reduced diversity of gut microbiota, elevated pro-inflammatory bacteria Prevotella and opportunistic pathogens Enterococcus, and reduced probiotic bacteria Bifidobacteria, among others, compared to healthy children (14). Hilty M et al. also suggested that the gut microbiota disorders in children with recurrent respiratory tract infections were significantly higher than those in children without recurrent respiratory tract infections and that children with recurrent respiratory tract infections may be affected by gut microbiota disorders, resulting in lower immunoglobulin levels, decreased immunity, and an increased risk of recurrent respiratory tract infections (15). Zhang D et al. showed that children with upper respiratory tract infections who were <3 years old and had an intestinal microecological imbalance had lower values of CD3+ and CD4+ than those in the non-disordered group and the healthy control group, and children with upper respiratory tract infections who had an imbalance of the gut microbiota at an age of less than 6 years old (including the <3 years old and the 3–6 years old age groups) had reduced levels of IgA compared with those of the non-disordered group and the healthy control group at the same age. It was demonstrated that gut microbiota disorders during respiratory tract infections affected both cellular and humoral immunity in children under 6 years of age (16), decreasing the gut microbiota-associated immune function of the children. See Figure 1, the gut microbiota dysbiosis during respiratory infections affects both immune and metabolic pathways.

## 3 Normal flora

The nasopharynx of infants is a sterile environment at birth, with contact with the external environment and the population, microorganisms gradually gather in the nasopharynx and other places in children, the respiratory microbiota is mainly thick-walled bacteria, actinomycetes, Aspergillus phylum, bacillus-like organisms, bacillus-like organisms, and Streptococcus aureus, etc., and the abundance and types of the flora tend to be stabilized when they grow up to about 6 years of age (3). A study of nasopharyngeal microecology in children before the age of 2 years showed that the composition of the dominant nasopharyngeal flora had already been formed by the age of 6 weeks, which could be any of the predominant species of Streptococcus spp., Moraxella catarrhalis, Staphylococcus spp., Corynebacterium spp., Mixed Corynebacterium spp., and/or Corynebacterium spp. (13) The pattern of nasopharyngeal flora gradually changed after the age of 6 weeks, e.g., the pattern of the predominant Staphylococcus spp. Disappeared gradually and shifted to a Haemophilus dominated, and the dominance of Corynebacterium spp.、Cunningham spp. was gradually replaced by Moraxella spp., Cunningham spp. The nasopharyngeal microecology was relatively stable if the nasopharyngeal flora was dominated by Moraxella spp. by 6 months of age. Nasopharyngeal microcosm with Haemophilus spp. and Streptococcus spp. Dominant was relatively more variable.

There were different correlations between different microorganisms as to whether or not they colonized: a positive correlation between Streptococcus pneumoniae colonization in the nasopharynx and Haemophilus influenzae; a negative correlation between C. catarrhalis, human rhinoviruses, and enteroviruses, and Staphylococcus aureus colonization; a positive correlation between C. catarrhalis colonization and coronaviruses and adenoviruses; a significant positive correlation between Staphylococcus aureus and influenza viruses; and between Haemophilus influenzae and human rhinoviruses, respiratory syncytial virus were positively correlated; there was a negative correlation between human rhinovirus and coronavirus. Nasopharyngeal colonization with Streptococcus pneumoniae was negatively correlated with Staphylococcus aureus (17, 18).

Recent studies have shown that the composition of oropharyngeal flora in children is similar to that of adults, and healthy adults have a rich flora settled in the oropharynx, including some pathogenic subgroups such as Streptococcus, Haemophilus influenzae, Neisseria spp. and possibly gram-negative anaerobic commensals such as Weilonella spp., Prevotella spp., Porphyromonas and Clostridium spp. However, there is a significant predominance of certain organisms in the children's oropharynx, e.g., Neisseria spp., Streptococcus granulosus spp., Prevotella, Porphyromonas and Clostridium spp. (19) A survey showed that healthy children aged 0–6 years were colonised by Haemophilus influenza, Streptococcus pneumoniae, Staphylococcus aureus, and Catamonas in the upper respiratory tract. With the growth of age, the colonisation of the upper respiratory tract increases (20). 2 to 3-year-old children's upper respiratory tract flora is very limited, and the diversity is low, 3 to 4-year-old children's upper respiratory tract in the diversity of species increased, and the ratio of aerobic bacteria and anaerobic bacteria is still 1:1.03, for this age of young children, the anaerobic and aerobic bacteria are the dominant species of the upper respiratory tract, and work together to maintain the microecological balance of the upper respiratory tract. Ecological balance of the upper respiratory tract. With the increase of age, the respiratory microecology tends to stabilize, and the ratio of respiratory aerobic and anaerobic bacteria in young people between the ages of 18 and 23 increased to 3.5:1 (21). Adult lung flora mainly from the oropharynx, the dominant genera include Streptococcus spp., Prevotella spp., Clostridium spp., and Weilonella spp., while Haemophilus spp. and Neisseria spp. are less common, with the thick-walled bacillus and anaplasmosis as the main composition, while the children's upper respiratory tract anatomical structure is different from that of the adults, and nasopharyngeal secretion is more, so the lung flora from the nasopharynx and the oropharynx, mainly from the upper respiratory tract, Molluscum contagiosum, Staphylococcus, Streptococcus, and Haemophilus (22). We have broadly summarised the composition of the main flora of the upper and lower respiratory tract in children as follows, see Table 1.

**Table 1:** Common commensal pathogens of the upper and lower respiratory tract.

Pathogenic commensal bacteria	conditionally pathogenic bacteria
upper respiratory tract	Streptococcus pneumoniae, Staphylococcus aureus, Haemophilus influenzae
lower respiratory tract	Streptococcus pneumoniae, Staphylococcus aureus, Catamorium spp.

## 4 Children's respiratory tract infection micro-ecological changes

Pathogens in pediatric RTIs may originate from vertical transmission, environmental reservoirs, or opportunistic overgrowth of resident flora (6), highlighting the complexity of microbial sources in disease progression. Most of the bacteria cultured from secretions during respiratory tract infections are commensal bacteria of the upper respiratory tract, and most of them show an increase in the number of pathogenic bacteria and a decrease in the number of pathogenic bacteria. The microecology of the nasopharynx changes during the development of acute respiratory infections. A cross-sectional study of Streptococcus pneumoniae colonization in children under 3 years of age found that the density of Streptococcus pneumoniae in the nasopharynx increased gradually before respiratory infection, peaked at the onset of respiratory infection, and then gradually returned to baseline levels at the time of non-infection during the later stages of respiratory infection (23). A study examined the differences in oropharyngeal flora between children with community-acquired pneumonia (CAP) and acute suppurative tonsillitis (AST) and healthy children. Differences in oropharyngeal flora between children with community-acquired pneumonia (CAP) and acute suppurative tonsillitis (AST) and healthy children were found: Streptococcus spp., Prevotella spp., Veillonella spp., Neisseria spp., Haemophilus spp., Cilioplasma spp., Porphyria spp. were the predominant genera in the CAP group, and Streptococcus spp., Prevotella spp., Veillonella spp., Neisseria spp., Porphyria spp. were the predominant genera in the AST group. Aeromonas, Neisseria, Porphyromonas, and Clostridium; and in the control group, the dominant genera were Streptococcus, Prevotella, Verrucella, Ciliophora, Neisseria, Porphyromonas, and Clostridium. Compared with healthy children, the oropharyngeal flora of children with respiratory tract infections had a change in the composition of some of the dominant genera, with an increase in the relative abundance of Streptococcus spp. and a decrease in the relative abundance of Megacoccus spp. and Campylobacter spp. in children with CAP; and a decrease in the relative abundance of ciliated bacteria spp. in the oral cavity of children with AST. Moreover, the abundance of oropharyngeal flora was significantly lower in children with pneumonia than in healthy children, while there was no significant change in the structure of oropharyngeal flora in children with AST, suggesting that lower respiratory tract infections may have a greater impact on the microecological balance of the oropharynx than upper respiratory tract infections (24). Common pathogens of respiratory tract infections in children are viruses, such as syncytial virus and influenza virus. During influenza virus infection, the respiratory microecological species change, and the number of bacteria colonizing the mucosal surface of the respiratory tract increases, leading to a greater chance of secondary infections and a series of differences in the clinical presentation of patients, such as higher C-reactive protein values, more frequent antibiotic treatment, and longer hospitalization cycles when S. aureus is combined with other bacteria compared to when S. aureus is solely colonized. Moreover, children are more likely to get pneumonia when Haemophilus influenzae, Moraxella catarrhalis, and Streptococcus pneumoniae are fixed than when S. aureus is fixed (20). Flora shift may also occur in respiratory tract infections, and it has been shown that children with upper respiratory tract infections have a higher detection rate of gram-positive organisms in throat swabs, with S. aureus being the most highly detected, and Escherichia coli being highly detected among the gram-negative organisms (25). Some nasopharyngeal colonization was detected in the secretions of children with pneumonia, demonstrating that nasopharyngeal colonization may occur in pneumonia.

## 5 Progress and prospect of probiotic-assisted treatment of children's respiratory tract infections

Now commonly used in the treatment of children's respiratory tract infections are antibiotics, but the extensive use of antibiotics can easily lead to the occurrence of drug resistance and gut microbiota dysbiosis (26), increasing the chances of recurrent respiratory infection. One study reported that multiple antibiotic exposures during a woman's pregnancy can cross the placenta to reach the unborn baby. This means that maternal antibiotic use during pregnancy may disrupt the balance of the infant's gut microbiota, leading to a decrease in beneficial flora and an increase in pathogenic bacteria. This imbalance in the microbiota may affect the production and transmission of neurotransmitters, which in turn may affect the infant's neurodevelopment. Thus this vertical transmission of antibiotics may increase the risk of respiratory disease in infants as well as affect their neurobehavioral status. In addition, there may be potential pathogens in the placenta, such as group B streptococci, which may carry antibiotic-resistant genes and pass through the placenta to the infant's microbiota (27). Based on the gut microbiota and respiratory diseases there is a reciprocal effect of the role of the gut microbiota, that the gut microbiota, especially the probiotic bacteria can be adjusted through the immune system to prevent and assist in the treatment of children's respiratory tract infections, to reduce the use of antibiotics. Prebiotics can also be added to assist in treatment when using probiotics, such as oligofructose and oligogalactose which can pass through the upper gastrointestinal tract without being digested, and arrive in the intestines to be fermented by the gut microbiota, providing energy and nutrition for the probiotics, thus increasing the number and activity of the probiotics and inhibiting the growth of the harmful bacteria, to maintain a balanced state of the gut microbiota. This balance is essential for intestinal health and can prevent the occurrence of many intestinal diseases, also beneficial in preventing gut microbiota disorders that tend to follow respiratory infections. A study found that Streptococcus aureus has an obvious antagonistic effect on pathogenic bacteria *in vitro* and *in vivo*, and the mouse model confirmed that Streptococcus aureus can inhibit the colonization of pathogenic bacteria in the mucosal epithelial cells, and protect the bacterial balance of the respiratory tract in mice. And type A streptococci such as Streptococcus salivarius, Streptococcus bradypneumoniae, Streptococcus oralis, etc. are the normal flora colonized in the upper respiratory tract of the human body, and dominate the oropharyngeal flora of healthy children, which is conducive to the protection of the respiratory tract and the prevention of infections (28). Relevant animal experiments have also found that mice treated with Streptococcus A can resist Streptococcus B infection and prevent respiratory infections, which needs to be further researched developed, and used in the prevention and treatment of respiratory infections in children (29). Studies have shown that probiotics can enhance the activation of inflammatory vesicles by SCFA, up-regulate the expression of IL-1β and IL-10, and activate the adaptive immune response to improve the body's ability to clear pathogenic bacteria and other pathogens, to achieve the positive regulation of the immunity of antipathogenic microorganisms, and it is believed that probiotics have a certain preventive and curative effect on COVID-19 infection (30). In clinical trials, it was also shown that children with upper respiratory tract infections taking Bifidobacterium bifidum had fewer occurrences of intestinal microecological imbalance, a lower average annual incidence of respiratory tract infections, and significantly fewer coughs, fever, secondary diarrhea, and antibiotics each time respiratory tract infections occurred, and laboratory tests showed that serum CRP and PCT concentrations in the children supplemented with probiotics were significantly lower than in the control group, and the serum IgG and IgA levels were significantly higher. Indicating that oral probiotics have a certain effect on preventing and treating recurrent respiratory tract infections in children and enhancing their immunity (31–33). Further supporting this, a recent meta-analysis by Zhang et al. demonstrated that probiotic supplementation significantly reduces the incidence and duration of respiratory infections in children, likely via gut microbiota modulation and mucosal immune enhancement (33).

## 6 Conclusion

Although the “lung-gut axis” is becoming better known, the respiratory microecology is complex and diverse, the intestinal microbiota is massive and the impact of microbiota changes on health still needs further research and exploration, and the general public has little knowledge of microbiota, which needs to be improved. Children's respiratory tract and intestinal tract have embryonic homology, and in the dynamic process of continuous development, the gut microbiota influences the occurrence and development of respiratory tract infections and other diseases through the regulation of humoral immunity and cellular immunity, and the displacement of the flora and changes in the number of species in respiratory infections also lead to gut microbiota disorders. Given the high prevalence of pediatric RTIs, their prevention and treatment have garnered significant attention. Based on the proposal of the “lung-intestinal axis”, the use of gut microbiota, the extraction of intestinal or respiratory probiotics to produce probiotic preparations for the prevention of respiratory tract infections, and adjuvant therapeutic drugs will become a new trend. Probiotics have shown beneficial effects not only in the prevention and control of gastrointestinal and respiratory infections but also have shown promise in the treatment of other diseases, see Table 2. However, further research is needed to elucidate probiotic therapies’ efficacy, adverse effects, and optimal timing for gut microbiota modulation in pediatric RTIs. As evidenced by clinical trials and meta-analyses (31–34), probiotics represent a promising strategy for respiratory infection prevention, though further research is needed to standardize protocols.

**Table 2:** Application of probiotic therapy.

Probiotics	Diseases prevented and assisted
Lactobacillus	Depression, Helicobacter pylori infection, intestinal tumours
Bifidobacterium, lactobacillus	Periodontitis, gingivitis, recurrent urinary tract infections, recurrent respiratory tract infections
Bifidobacterium, lactobacillus, saccharomycetes	Diarrhoea, irritable bowel syndrome, ulcerative colitis, hepatic encephalopathy, functional gastrointestinal disorders
Streptococcus oralis	Respiratory tract infections
